# The regulatory roles of T helper cells in distinct extracellular matrix characterization in breast cancer

**DOI:** 10.3389/fimmu.2022.871742

**Published:** 2022-09-08

**Authors:** Qi Tian, Huan Gao, Yingying Ma, Lizhe Zhu, Yan Zhou, Yanwei Shen, Bo Wang

**Affiliations:** ^1^ Department of Radiology, the First Affiliated Hospital of Xi’an Jiaotong University, Xi’an, China; ^2^ Department of Medical Oncology, the First Affiliated Hospital of Xi’an Jiaotong University, Xi’an, China; ^3^ Department of Breast Surgery, the First Affiliated Hospital of Xi’an Jiaotong University, Xi’an, China; ^4^ Department of Surgery Oncology, Shaanxi Provincial People’s Hospital, Xi’an, China; ^5^ Center for Translational Medicine, the First Affiliated Hospital of Xi’an Jiaotong University, Xi’an, China

**Keywords:** extracellular matrix, immune cell infiltration, T helper cells, prognostic model, breast cancer

## Abstract

**Background:**

Tumors are characterized by extracellular matrix (ECM) remodeling and stiffening. The ECM has been recognized as an important determinant of breast cancer progression and prognosis. Recent studies have revealed a strong link between ECM remodeling and immune cell infiltration in a variety of tumor types. However, the landscape and specific regulatory mechanisms between ECM and immune microenvironment in breast cancer have not been fully understood.

**Methods:**

Using genomic data and clinical information of breast cancer patients obtained from The Cancer Genome Atlas (TCGA) and Gene Expression Omnibus (GEO) databases, we conducted an extensive multi-omics analysis to explore the heterogeneity and prognostic significance of the ECM microenvironment. Masson and Sirius red staining were applied to quantify the contents of collagen in the ECM microenvironment. Tissue immunofluorescence (IF) staining was applied to identify T helper (Th) cells.

**Results:**

We classified breast cancer patients into two ECM-clusters and three gene-clusters by consensus clustering. Significant heterogeneity in prognosis and immune cell infiltration have been found in these distinct clusters. Specifically, in the ECM-cluster with better prognosis, the expression levels of Th2 and regulatory T (Treg) cells were reduced, while the Th1, Th17 and T follicular helper (Tfh) cells-associated activities were significantly enhanced. The correlations between ECM characteristics and Th cells infiltration were then validated by clinical tissue samples from our hospital. The ECM-associated prognostic model was then constructed by 10 core prognostic genes and stratified breast cancer patients into two risk groups. Kaplan-Meier analysis showed that the overall survival (OS) of breast cancer patients in the high-risk group was significantly worse than that of the low-risk group. The risk scores for breast cancer patients obtained from our prognostic model were further confirmed to be associated with immune cell infiltration, tumor mutation burden (TMB) and stem cell indexes. Finally, the half-maximal inhibitory concentration (IC50) values of antitumor agents for patients in different risk groups were calculated to provide references for therapy targeting distinct ECM characteristics.

**Conclusion:**

Our findings identify a novel strategy for breast cancer subtyping based on the ECM characterization and reveal the regulatory roles of Th cells in ECM remodeling. Targeting ECM remodeling and Th cells hold potential to be a therapeutic alternative for breast cancer in the future.

## Introduction

Breast cancer is the most common cancer in the world (1). The occurrence of tumor invasion and metastasis is an important risk factor affecting the prognosis of breast cancer patients. Therefore, it is of great clinical significance to further explore the mechanism of breast cancer invasion and metastasis and identify new therapeutic targets against tumor metastasis. For the past few years, the significance of the interaction between tumor cells and tumor microenvironment (TME) in malignant tumor progression has attracted extensive attention (2, 3). As a key component of TME, extracellular matrix (ECM) mainly includes collagen, fibronectin, laminin, glycosaminoglycan, proteoglycan and various ECM remodeling enzymes, whose main function is to provide important biochemical and biomechanical support for the cells in it. ECM remodeling, that is the significant changes in ECM contents and arrangement, has been shown to be closely associated with the differentiation, proliferation and metastasis of tumors (4, 5). A study of pan-cancer landscape of ECM gene dysregulation found that a subset of ECM genes specifically dysregulated in tumors, and high expression of this subset genes was adversely prognostic in pan-cancer analyses (6). Extensive alterations of the ECM have been found in breast cancer, including the upregulation or altered arrangement of fibrillar collagen, fibronectin and other remodeling enzymes, and the consequent enhancement of ECM stiffness (7–9). There is also extensive clinical imaging and pathological evidence that the denseness and hardness of tumor tissue are closely related to its malignancy (10). During the development of mammary cells from normal to ductal carcinoma *in situ* and then invasive carcinoma, the hardness of ECM increases gradually (11). Recent studies have shown that increased tissue stiffness in breast cancer promotes the cytoskeleton remodeling by activating the Rho/ROCK signaling pathway, and parallel aligned collagen can promote epithelial-mesenchymal transition (EMT) by upregulating TGF-β receptors, accelerating invasion and metastasis (12, 13). In addition, ECM remodeling can also cause overexpression of EGFR, ERBB2, CD44 and other receptors in TME, and further induce tumor invasion and metastasis through the transactivation of downstream PI3K/Akt, MAPK and other signaling pathways (13–15).

The interrelationship between immune cells and ECM, both of which are important components consisting of TME, has become an emerging and crucial topic in the field of tumor biology. The maintenance of a balance between immune system and ECM remodeling dynamics is essential to ensure the homeostasis of physiological processes. Immune cells are involved in the degradation, synthesis and reconstruction of ECM components (16, 17). Several ECM components, such as laminin and collagen, can be served as ligands for immune cells, so as to facilitate the adhesion and trafficking process (18). The dynamic crosstalk between ECM and immune cells could alter the TME from a tumor suppressive to tumorigenic state. For example, this process accelerates the transformation of tumor-associated macrophages (TAMs) from pro-inflammatory M1 macrophages to anti-inflammatory M2 macrophages, which further promotes tissue remodeling and tumor progression (19). In addition, the complex T helper (Th) cells-mediated immune response has also been reported to be closely linked to the accumulation of collagen and other ECM components (20). In skin cancer, chemokines such as CCL-17 and CCL-18 secreted by stromal cells can recruit immunosuppressive regulatory T (Treg) cells to infiltrate in tumor islands, and overexpression of CCL-17 and its ligand CCR-4 can promote the aggregation of CD4-positive (CD4+) Treg, Th2 and Th17 cells (21, 22). In summary, the ECM in tumor is highly disorganized and remodeled due to the abnormal activation of fibroblasts and aberrant deposition of ECM components by the interaction of tumor, stromal and immune cells. Therefore, revealing the interplay between ECM and immune system is critical for fully understanding the significance of ECM remodeling in cancer progression, and rational designing new strategies for immunotherapy. However, there is still a lack of comprehensive and integrated analysis of ECM-related gene expression profiles in tumors, especially in breast cancer, as well as the lack of correlation analysis between distinct ECM signatures and immune-related features including Th cell subpopulations.

In this present study, we questioned whether breast cancer has heterogeneous ECM remodeling-related features and which signaling pathways and biological processes drive the formation of these phenotypes? With multi-omics data from publicly available databases, we classified breast cancer patients into distinct ECM-related clusters, and revealed significant differences in the abundance of immune cells, especially Th cells, within these distinct ECM-related subpopulations. The above correlation results between ECM and Th cells were also validated by collecting clinical tissue samples. In addition, we developed and validated the ECM-related prognostic signature using RNA-seq and prognostic information from breast cancer cohorts of public databases. Our systematic analysis of the ECM characteristics in breast cancer will provide an important research reference for prognostic evaluation and treatment strategy formulation.

## Materials and methods

### Patients and datasets

A total of 1727 samples from The Cancer Genome Atlas (TCGA) cohort and 3 Gene Expression Omnibus (GEO) cohorts were included in this study. The RNA-Seq expression profile of 1208 samples, including 1096 breast cancer samples and 112 normal mammary samples, mutation data, copy number variation data and the corresponding clinical information of 1085 samples were downloaded from TCGA database (https://portal.gdc.cancer.gov). In addition, the RNA-seq data and follow-up information of 3 external validation cohorts were downloaded from GEO database (http://www.ncbi.nlm.nih.gov/geo), including 327 samples of GSE20685 (23), 104 samples of GSE42568 (24), and 88 samples of GSE20711 (25). A total of 30 cases of breast cancer tissues used to validate the link between ECM characteristics and Th cells infiltration were collected from the First Affiliated Hospital of Xi’an Jiaotong University. All the patients had signed the informed consent before surgery, and the present study was authorized by the Ethics Committee of the First Affiliated Hospital of Xi’an Jiaotong University, and followed TCGA and GEO data access policies and publication guidelines.

### Consensus clustering

Subsequently, 328 ECM-associated genes were obtained based on gene ontology terms, which were attached in **Supplementary Table 1**. According to the expression levels of the above ECM-associated genes, the ConsensusClusterPlus package in R software was applied to perform consensus clustering to determine the optimal clusters for breast cancer patients in TCGA database. The results of K-means clustering from k=2 to k=9 were represented by heatmaps, and the optimal cluster number was determined by the consistent cumulative distribution function (CDF) plot and the area of delta region.

### Identification of differentially expressed genes and functional enrichment analysis

The DEGs between breast cancer and normal mammary gland tissues, as well as between different ECM-clusters, were all identified by the limma R package. The Wilcoxon test was used to identify DEGs, and genes with log_2_|fold change (FC) |> 1 and false discovery rate (FDR) < 0.05 were termed as DEGs. Gene Ontology (GO) and Kyoto Encyclopedia of Genes and Genomes (KEGG) functional enrichment analyses were then employed to identify potential biological functions and signaling pathways of DEGs using the clusterProfiler package of R software.

### Abundance calculation of immune cells, fibroblasts and TME scores

The single-sample gene set enrichment analysis (ssGSEA) algorithm was used to calculate the enrichment levels of 23 immune cells, and the gene sets corresponding to these 23 immune cells were summarized in **Supplementary Table 2**. The ssGSEA algorithm was implemented through GSVA package. The relative abundance of fibroblasts was obtained by the Microenvironment Cell Populations-counter (MCP-counter) method. The deconvolution algorithm CIBERSORT was also applied to impute the degree of immune cells infiltration from the bulk tumor transcriptome data. A total of 1000 simulations were conducted for CIBERSORT, and samples with *p* < 0.05 were selected for the subsequent analysis. The stromal scores, immune scores and ESTIMATE scores were then measured using ESTIMATE package of R software, an algorithm estimating the tumor purity as well as the degree of immune and stromal infiltration based on RNA-seq data from tumor samples containing TME components.

### Tissue collagen staining and quantification

For Masson staining (Solarbio), paraffin embedded sections from breast cancer sample tissues were stained according to the manufacturer’s instructions to examine the contents and arrangement of collagen. After staining, the green or blue colored parts were fibrillar collagens, and red parts were muscle fibers. Picrosirius red analysis was achieved by using paraffin sections of breast cancer tissues stained with the combined Sirius Red/Fast Green dye solution (Chondrex), in which Sirius Red specifically bind to the helical structure of fibrillar collagens and Fast Green bind to non-collagenous proteins in tissues. Whole stained sections were scanned with the slide scanner (Leica) at 20× magnification. For the collagen quantification results of Masson staining for each sample, 3 fields of view were randomly selected from the slices for the following statistical analysis, and collagen contents in breast cancer tissues were quantified by ImageJ software.

### Tissue immunofluorescence staining

The paraffin embedded sections from breast cancer tissue samples were deparaffinized in xylene and dehydrated in gradient concentration of ethanol. Antigen repair of tissue slides was subsequently performed in ethylene diamine tetraacetic acid (EDTA) buffer (pH 8.0) for 15 min in a microwave oven, followed by permeabilized with 0.5% TritonX-100 for 10 min, and blocked with 5% bovine serum albumin (BSA). Then tissue slides were incubated overnight with specific primary antibodies, followed by secondary antibodies of Cy3-conjugated goat anti-rabbit IgG and FITC-conjugated goat anti-mouse IgG(Proteintech). Primary antibodies used for IF staining included CD4 (Proteintech, 67786, 1:200), T-bet (Abcam, ab150440, 1:30), CCR4 (Abcam, ab59550, 1:50), CD25 (Abcam, ab231441, 1:50) and IL-17A (Abcam, ab79056, 1:100). DAPI solution (Bioworld, 1:1000) was next used for nuclear staining. Image capture was performed by a Leica confocal microscope at 40× magnification. For the quantitative analysis of each Th cells, we randomly selected 3 fields from the slices of each sample for statistical analysis.

### Establishment of the ECM -associated prognostic model

Univariate COX regression analysis by survival R package was used to identify the potential prognostic DEGs between different ECM-clusters. The prognostic genes with *p*<0.05 filtered by univariate COX regression were subsequently included in the least absolute shrinkage and selection operator (LASSO) regression analysis to determine the core prognostic genes by glmnet package. The risk score for each breast cancer patient in our prognostic model was calculated using this following formula: risk score = β1*expG1 + β2*expG2 + … + βn*expGn, where β was the regression coefficient of the genes obtained by LASSO regression and expG was the expression level of core prognostic gene. The formula was then applied to the external GEO validation cohorts to verify the validity and reproducibility of our prognostic model. Combining the risk scores with other clinical parameters, we then constructed a nomogram prediction model by independent risk factors obtained by multivariable COX regression using the R rms package.

### Prediction of the drug sensitivity of patients in different risk groups

The pRRophetic package constructs ridge regression model based on Genomics of Drug Sensitivity in Cancer (GDSC) cell line expression profiles and TCGA gene expression profiles to predict the half-maximal inhibitory concentration (IC50) of antitumor agents. To identify potential therapeutic agents for breast cancer patients with different ECM characteristics, we applied the pRRophetic package to predict the IC50 of chemotherapeutic agents for breast cancer patients in different risk groups.

### Statistical analysis

All data were analyzed using R version 4.1.0 or GraphPad Prism 8, and all experiments were repeated at least 3 times. These results were presented as mean ± standard deviation (SD). Student’s two-sided t-test was used to compare the differences between two groups. Survival between different risk groups were compared by Kaplan-Meier curves followed by log-rank test. Correlation analysis was conducted by pearson’s test. *P* < 0.05 was considered as statistically significant.

## Results

### The landscape of expression levels, mutation and copy number variation of ECM-related genes in breast cancer

Initially, a total of 328 ECM-related genes were curated based on gene ontology terms for this study. Subsequently, we identified 118 ECM-related DEGs between breast cancer and normal mammary gland tissues of TCGA database by the criteria of log_2_|FC| > 1, FDR < 0.05, of which 59 genes were up-regulated while the other 59 genes were down-regulated in tumor tissues. The heatmap in **Supplementary Figure 1A** and volcano map in **Supplementary Figure 1B** depicted the expression levels and distribution of the above ECM-associated DEGs. In addition, we also extracted the mutation and CNV data of ECM -related genes from TCGA database. From the waterfall plot, we observed that in 986 breast cancer patients with mutation information, a total of 624 patients (63.29%) had mutations in ECM -related genes. We showed the genes with the top 30 mutation rates in **Supplementary Figure 1C**, among which the mutation rates of CDH1, a crucial gene involved in the formation of cell adhesion junction, was the highest (13%). Other genes with high mutation frequency included neurofibromatosis type 1-related gene NF1 (4%), several collagen molecules such as COL12A1 (2%), COL6A3 (2%), COL14A1 (2%), and the basement membrane-related molecules LAMA1 (2%), LAMA2 (2%). **Supplementary Figure 1D** demonstrated the CNV frequencies of ECM-related genes, where ICAM2, PECAM1, SOX9 and ITGB4 had high copy number gain frequencies, while MFAP2, HSPG2 and PDPN had high copy number loss frequencies. The above results suggested that there was significant heterogeneity in genomic alterations and expression levels of ECM -related genes in breast cancer.

### ECM -related phenotypes in breast cancer

**Figure 1:**
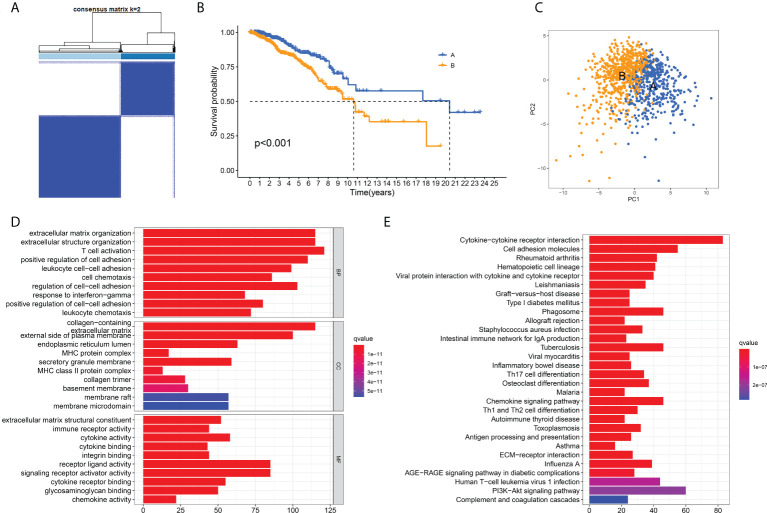
Identification of ECM-clusters in breast cancer based on ECM-associated genes. **(A)** Breast cancer patients in TCGA database were stratified into two different ECM-clusters according to consensus clustering with k=2. **(B)** Kaplan-Meier survival analysis demonstrated the prognostic differences between these two ECM-clusters. **(C)** Dimensionality reduction algorithms of PCA showed that these two ECM-clusters were separately distributed. **(D)** GO analysis between different ECM-clusters demonstrated the differences in terms of biological processes, cellular components and molecular functions. **(E)** KEGG analysis demonstrated the differential expression of signaling pathways among ECM-clusters. ECM, extracellular matrix; TCGA, the Cancer Genome Atlas; PCA, principle component analysis; GO, Gene Ontology; KEGG, Kyoto Encyclopedia of Genes and Genomes.

To further clarify the characteristics of ECM in breast cancer, we analyzed the expression profiles of 328 ECM -related genes in 1208 breast cancer patients in TCGA database to develop consensus clustering. According to the CDF in **Supplementary Figure 2A** and the function delta area in **Supplementary Figure 2B**, we chose to divide breast cancer patients into two clusters for the clustering outcome was stable when k=2 (**Figure 1A**). Next, we compared the overall survival (OS) data of these two different ECM-clusters and found that the patients in cluster A lived longer than patients in cluster B (**Figure 1B**). Dimensionality reduction algorithm of PCA was then used to confirm the samples of the above two clusters were separately distributed (**Figure 1C**). In order to explore the underlying mechanism accounting for the prognosis differences between distinct ECM-clusters, we then analyzed the DEGs between cluster A and cluster B by the cutoff value of log2|FC| > 1 and FDR < 0.05 and identified a total of 1214 DEGs. Functional enrichment analysis of GO and KEGG of DEGs were subsequently performed, and we were surprised to find that in addition to the differences in ECM-associated pathways, there were also significant differences in several immune-related pathways between these two ECM-clusters. For example, GO analysis demonstrated that DEGs were significantly enriched in biological processes (BP) related with T cell activation and cell chemotaxis (**Figure 1D**). For molecular function (MF), significant differences in immune system-related activities (such as immune receptor activity, cytokine activity and chemokine activity) were also observed (**Figure 1D**). The bar plot of **Figure 1E** demonstrated the significantly enriched KEGG pathways, in particular, the significant differences in the cellular differentiation process of Th (including Th1, Th2 and Th17) cells could be found.

### Characteristics of immune cell infiltration in distinct ECM-clusters

**Figure 2:**
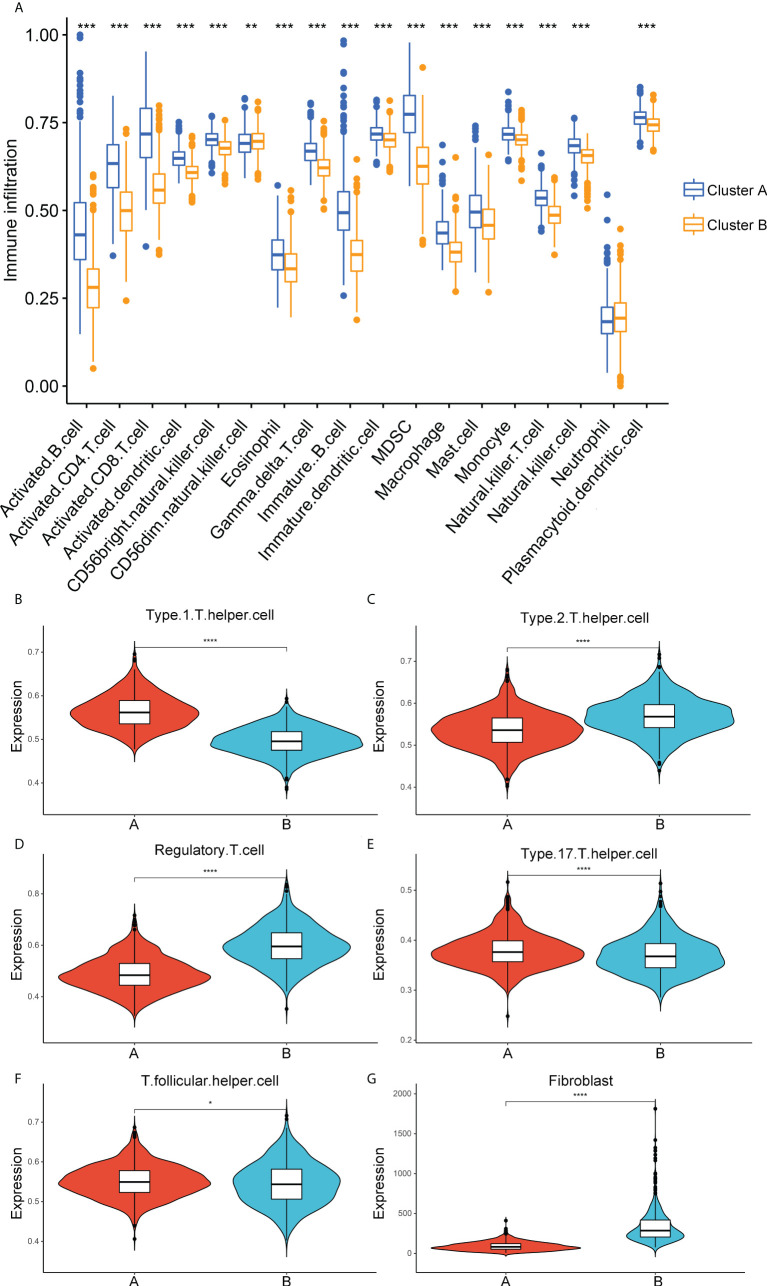
Correlation analysis between immune cell infiltration and ECM-clusters. **(A)** Distribution of the relative abundance of 18 immune cells obtained by ssGSEA algorithm in different ECM-clusters. B-F. Relative contents of Th cell subpopulations, including Th1 **(B)**, Th2 **(C)**, Treg **(D)**, Th17 **(E)** and Tfh **(F)** cells in different ECM-clusters. **(G)** Relative abundance of fibroblasts in different ECM-clusters calculated by MCP-counter algorithm. ssGSEA, single-sample gene set enrichment analysis; Th cell, T helper cell; Th1, type 1 T helper cell; Th2, type 2 T helper cell; Treg, regulatory T cell; Th17, type 17 T helper cell; Tfh, T follicular helper cell; MCP-counter, Microenvironment Cell Populations-counter; *p<0.05; **p<0.01; ***p<0.001; ****p<0.0001.

Considering the significant enrichment of immune pathways between distinct ECM-clusters, we next applied ssGSEA algorithm to measure the relative contents of 23 immune cells in breast cancer patients from TCGA database. For the majority of immune cells, such as activated CD8-positive (CD8+) T cells and natural killer (NK) cells with tumor-killing effects, their relative contents in cluster A were significantly higher than those in cluster B (**Figure 2A**). However, the relative abundance of CD56dim-NK cells in cluster A was lower than that in cluster B, and there was no significant difference in the contents of neutrophils between these two ECM-clusters. In addition, we also noticed that the distribution of Th cell subpopulations in the two ECM-clusters was significantly different (**Figure 2A**). Among them, the pro-inflammatory Th1 cells were highly expressed in cluster A, and anti-inflammatory Th2 and Treg cells were expressed at low levels in cluster A, indicating that the good prognosis of cluster A may be associated with the different differentiation routes and maintenance status of Th cell subpopulations induced by distinct ECM characteristics in breast cancer (**Figures 2B–D**). For other Th cell subsets, the results calculated by ssGSEA algorithm also showed that the proportions of Th17 and T follicular helper (Tfh) cells in cluster A were higher than those in cluster B, although the differences were not as significant as those in Th1, Th2 and Treg cells (**Figures 2E, F**). Moreover, the MCP-counter algorithm was also used to calculate the relative abundance of intra-tumoral fibroblasts, and the results showed that the fibroblasts abundance in cluster B was much higher than that in cluster A, suggesting that high fibroblast content was associated with the state of intra-tumoral immunosuppression as well as poor prognosis for breast cancer patients (**Figure 2G**).

### Validation of Th cells infiltration characteristics by clinical tissue samples

**Figure 3:**
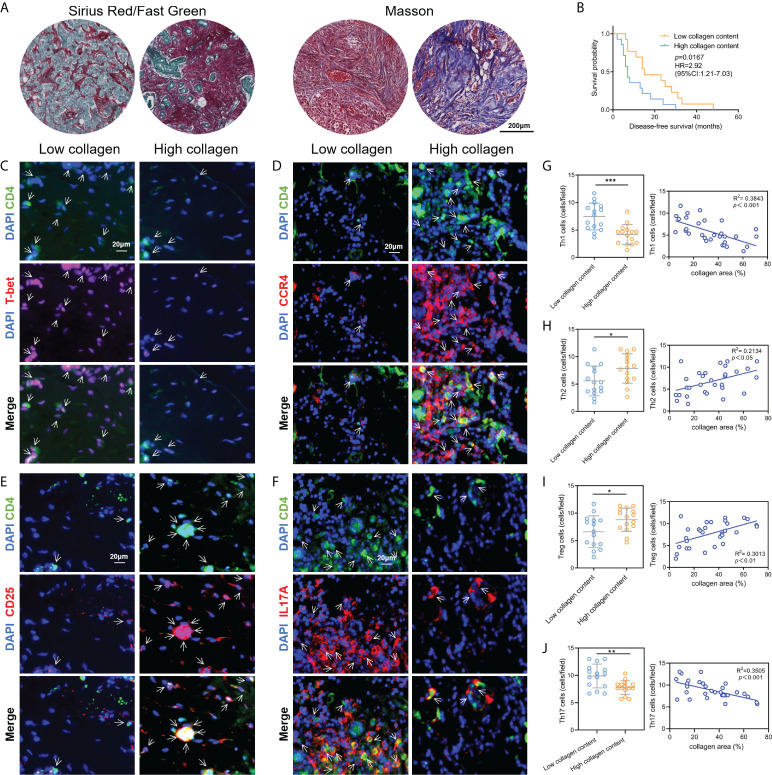
Validation of the correlation between ECM characteristics and Th cell infiltration by clinical tissue samples. **(A)** Measurements of collagen contents in breast cancer tissues by Sirius red staining and Masson staining. **(B)** Kaplan-Meier survival analysis revealed the DFS differences in breast cancer patients with different collagen contents. C-F. Tissue immunofluorescence staining showed the contents of Th1 **(C)**, Th2 **(D)**, Treg **(E)** and Th17 **(F)** cells in breast cancer tissue samples. G-J. Contents of Th1 **(G)**, Th2 **(H)**, Treg **(I)** and Th17 **(J)** in breast cancer tissues with different collagen abundances, and the corresponding correlation analysis between collagen abundances and Th cell contents. DFS, diseases-free survival; *p<0.05; **p<0.01; ***p<0.001.

Subsequently, to validate the above correlation results between ECM and Th cells infiltration obtained from the public database, a total of 30 breast cancer tissue samples were collected and validated by collagen staining and IF labeling of Th cells’ markers. We applied Masson staining and Sirius red staining to determine the contents of collagen in breast cancer tissue samples and quantified the percentage of collagen contents using ImageJ software (**Figure 3A**). According to the collagen contents, these 30 patients were divided into two groups: high collagen content and low collagen content. The analysis of disease-free survival (DFS) data showed that the DFS of patients with low collagen content was significantly prolonged compared with patients with high collagen content (**Figure 3B**). Afterwards, tissue IF staining was used for co-labeling CD4 with T-bet, CCR4, CD25 and IL17A to label the contents of Th1, Th2, Treg and Th17 cells, respectively, where CD4 was taken as a pan-T helper cell marker (**Figures 3C-F**). Statistical analysis demonstrated that the abundance of Th1 and Th17 cells were significantly higher in the low collagen content group than in the high collagen group, while Th2 and Treg cells in the high collagen group were significantly higher than those in the low collagen group. Correlation analysis also showed that Th1 and Th17 cell contents were negatively correlated with collagen contents, while Th2 and Treg cell contents were positively correlated with collagen contents (**Figures 3G–J**). The above results from clinical tissue samples further validate the link between ECM characteristics and Th cell subpopulations.

### Identification of distinct gene-clusters using prognosis-related DEGs

**Figure 4:**
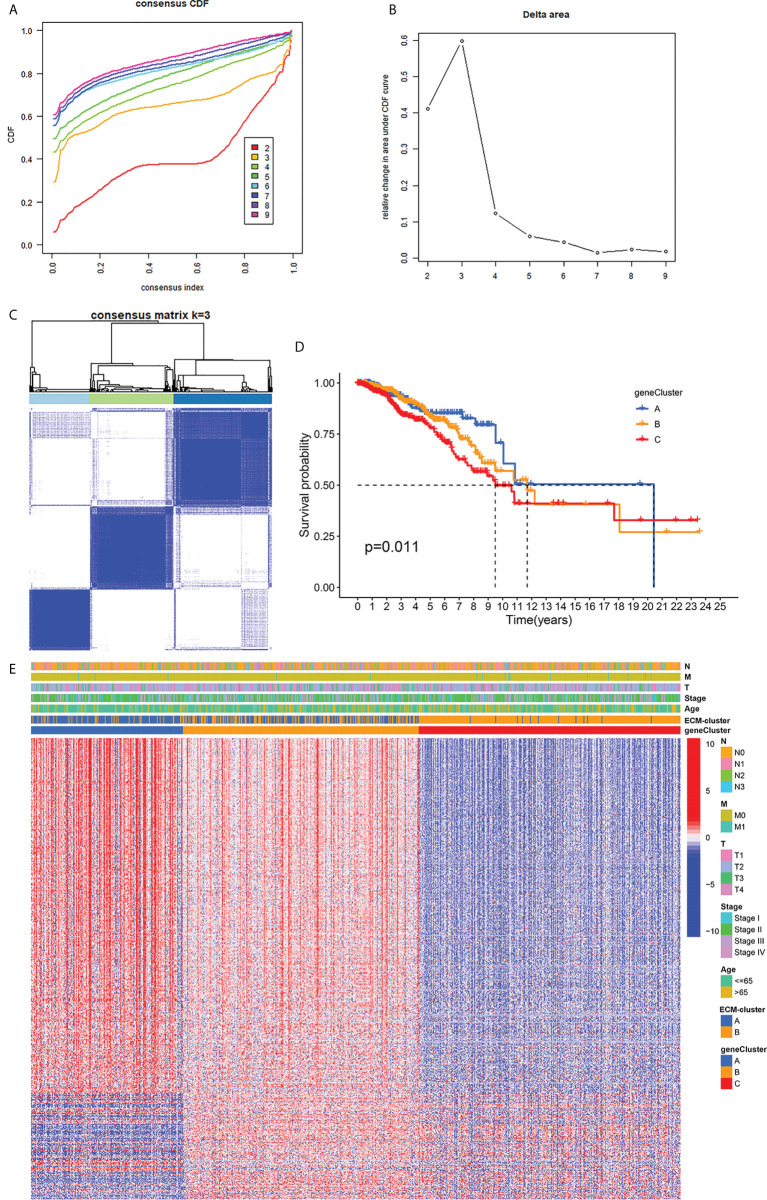
Classification of breast cancer patients into three gene-clusters based on DEGs between ECM-clusters. **(A)** The CDF curves obtained by consensus clustering based on the DEGs between ECM-clusters. **(B)** The function delta area under CDF curves. **(C)** Classification of breast cancer patients into three gene-clusters according to consensus clustering with k=3. **(D)** Kaplan-Meier survival analysis demonstrated the prognostic differences between these three gene-clusters. **(E)** The heatmap demonstrated the overview of DEGs in the ECM-clusters and gene-clusters. DEGs, differential expressed genes; CDF, cumulative distribution function.

We next performed univariate COX regression analysis on the DEGs between the two distinct ECM-clusters and extracted the expression levels of prognosis-associated DEGs. Based on the above prognosis-associated DEGs, we performed consensus clustering again and chose to classify breast cancer patients into three gene-clusters according to the CDF and function delta area (**Figures 4A–C**). The results of Kaplan-Meier survival analysis also showed significant differences in OS among these three different gene-clusters (**Figure 4D**). The heatmap in **Figure 4E** demonstrated the overview of DEGs in the ECM-clusters and gene-clusters, suggesting that there were significant differences in gene expression patterns between distinct clusters.

### Development and validation of the ECM-related prognostic model

**Figure 5:**
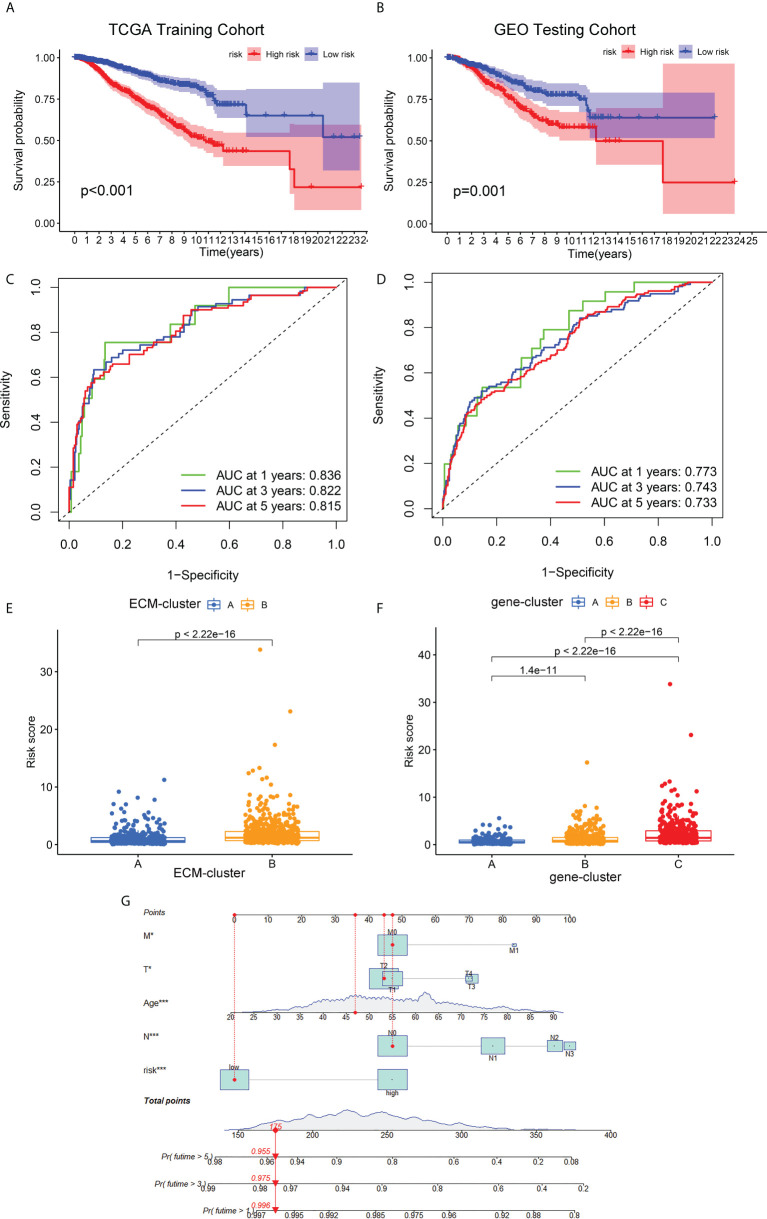
Development and validation of the ECM-associated prognostic model for breast cancer patients. **(A)** Kaplan-Meier survival analysis showed the OS differences of breast cancer patients in different risk groups from TCGA training cohort. **(B)** Kaplan-Meier survival curve of OS of breast cancer patients from GEO testing cohort. **(C)** AUC of time-dependent ROC curves to evaluate the predictive efficacy of the prognostic model in TCGA training cohort. **(D)** AUC of time-dependent ROC curves to evaluate the predictive efficacy of the prognostic model in GEO testing cohort. **(E)** Distribution of risk scores of breast cancer patients in different ECM-clusters. **(F)** Distribution of risk scores of breast cancer patients in different gene-clusters. **(G)** Nomogram for predicting 1-, 3- and 5-year survival of breast cancer patients constructed by combining risk scores and clinical parameters. OS, overall survival; GEO, Gene Expression Omnibus; AUC, area under the curve; ROC, receiver operating characteristic curve; *p<0.05; ***p<0.001.

The prognostic ECM -associated DEGs of breast cancer patients were then used for the following LASSO Cox regression analysis. According to the optimal penalty parameter (λ) shown in the **Supplementary Figures 3A, B**, the ECM -related prognostic model based on 10 core prognostic genes was established. The risk scores of breast cancer patients in this prognostic model can be obtained by this following formula: Risk score = 0.167 * expression level of P4HA3 + 0.297 * expression level of ZMAT3 +(-0.168) * expression level of TNN + 0.328 * expression level of ENPEP + 0.260 * expression level of PCDHB12 + (-0.298) * expression level of SGCE + (-0.275) * expression level of PDLIM4 + 0.170 * expression level of WNT7B + (-0.146) * expression level of FGD3 + (-0.122) * expression level of IL33. Then, the breast cancer patients could be stratified into two different risk groups by the median value of risk scores, and the patients in the high-risk group had a higher death probability than those in the low-risk group (**Figure 5A**). Next, the RNA-seq data and prognostic information from 3 GEO cohorts (GSE20685, GSE20711 and GSE42568) of breast cancer patients were extracted for external validation of the stability and reproducibility of our prognostic model. This formula obtained from the TCGA training cohort was then applied to calculate the risk scores of breast cancer patients from the GEO validation cohort, and Kaplan-Meier analysis also showed that the prognosis of the high-risk group was significantly worse than that of the low-risk group (**Figure 5B**). Furthermore, the predictive efficacy of our prognostic model for predicting OS for breast cancer patients was assessed by time-dependent receiver operating characteristic (ROC) curves, and the results showed that our prediction model could achieve superior fitting effect both in the TCGA training cohort and the GEO validation cohort (**Figures 5C, D**). We then detected the link between risk scores and distinct ECM-clusters or gene-clusters, and found that the risk scores were much higher in the clusters with poor prognosis (**Figures 5E, F**). Afterwards, we incorporated the clinical parameters and risk scores into the establishment of nomogram model. The corresponding scores of each variable can be obtained from top scale based on the coefficient values from Cox regression, and the total summed scores of each breast cancer patient can be used to estimate the 1-, 3- and 5-year survival probability (**Figure 5G**).

### Correlation analysis of ECM-related prognostic risk with immune infiltration, tumor mutation burden and drug sensitivity

**Figure 6:**
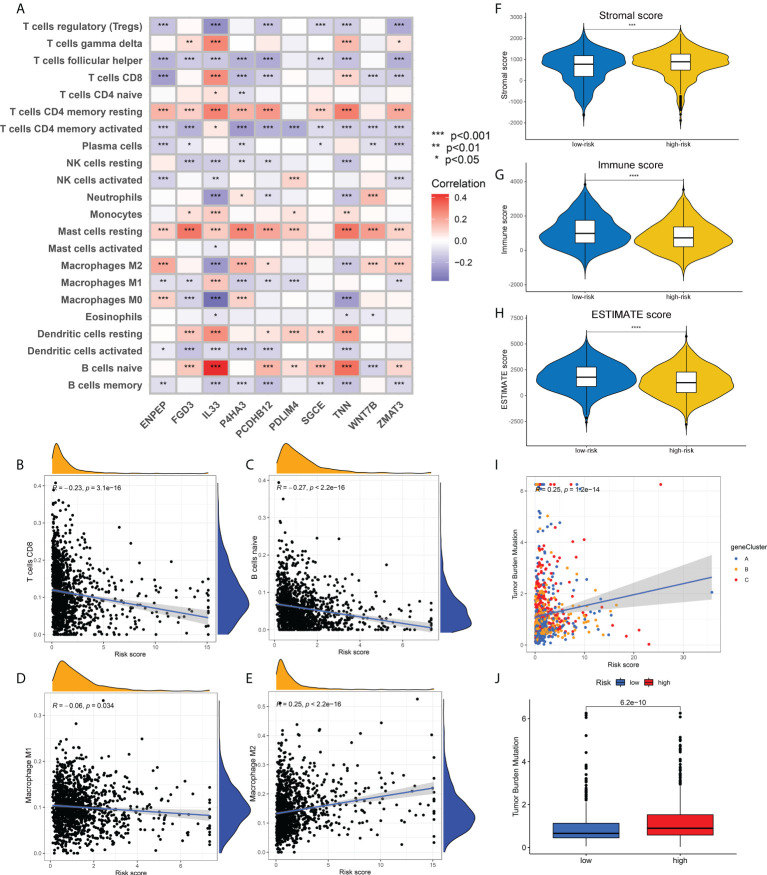
Association of risk scores obtained from the prognostic model with immune infiltration and TMB. **(A)** Heatmap showing the correlation between the relative abundance of 22 immune cells and 10 core prognostic genes of our prognostic model. **(B-E)**. Correlation analysis between risk scores and the contents of CD8+ T cells **(B)**, naïve B cells **(C)**, M1 macrophages **(D)** and M2 macrophages **(E)**. **(F-H)**. The distribution differences in stromal scores **(F)**, immune scores **(G)** and ESTIMATE scores **(H)** in breast cancer patients of different risk groups. **(I)** Correlation analysis between risk scores and TMB of breast cancer patients. **(J)** The distribution differences of TMB in breast cancer patients from different risk groups. TMB, tumor mutation burden; *p<0.05; **p<0.01; ***p<0.001; ****p<0.0001.

To verify the correlation between our prognostic model and intra-tumoral immune cell infiltration characteristics, we next applied CIBERSORT algorithm to calculate the relative abundance of 15 immune cells. The heatmap in **Figure 6A** demonstrated that all the 10 core prognostic genes constituting the prognostic model significantly correlated with the expression levels of immune cells. The correlation analysis between risk scores and immune cells showed that, the relative abundance of CD8+ T cells, naïve B cells, and M1 macrophages were negatively correlated with patients’ risk scores (**Figures 6B–D**), while the tumor-promoting M2 macrophages were positively correlated with risk scores (**Figure 6E**). To confirm the accuracy of the link between the prognostic model and TME, ESTIMATE algorithm was also applied to measure the stromal scores, immune scores and ESTIMATE scores for breast cancer patients. We found that compared with the patients in the low-risk group, the stromal scores in the high-risk group were significantly up-regulated (**Figure 6F**), while the immune scores and ESTIMATE scores were significantly down-regulated (**Figures 6G, H**).

Correlation analysis also showed a positive correlation between TMB and risk scores, that is, the TMB levels in patients with high-risk were higher than those in patients with low-risk (**Figures 6I, J**). **Supplementary Figures 4A, B** depicted the mutation profiles of the high-risk and low-risk groups, respectively. The mutation frequency of patients in the high-risk group was slightly higher than that in the low-risk group, but the most common mutated genes were almost identical. Correlation analysis of risk scores and stem cell index also demonstrated a positive correlation between them (**Supplementary Figure 4C**). Although this correlation was not strong enough, the relationship between ECM characteristics and stem cell-like properties still needs to be further investigated. Finally, we compared the differences in drug sensitivity between the high-risk group and the low-risk group and screened that the IC50 values for Bicalutamide and CMK were lower in the high-risk group (**Supplementary Figures 4D, E**), while the IC50 values for Gefitinib and Gemcitabine were lower in the low-risk group (**Supplementary Figures 4F, G**). The above drug sensitivity results are expected to provide references for targeted treatment strategies for breast cancer patients with distinct ECM characteristics.

## Discussion

The ECM consists of a variety of structural macromolecules, such as collagen, laminin, fibronectin and elastin, which stores growth factors and bioactive molecules, including matrix metalloproteinases (MMPs), heparan sulfate, and fibroblast growth factors (26). The tumor and stromal cells in the TME secrete interstitial matrix to support cell proliferation and tumor growth. In particular, the ECM components at the tumor site provide the structural foundation for biological activities, allowing tumor growth, motility and differentiation (27). Breast cancer patients stratified according to ECM components and arrangement demonstrated significantly distinct prognoses, among which the ECM characteristics of patients with the worst prognosis were characterized by ordered collagen arrangement and high expression of integrins and MMPs (6). The ECM-associated gene set was extracted based on gene ontology terms, and we then classified breast cancer patients into two ECM-clusters according to the ECM genes expression profiles. Significant prognostic difference was found between these two ECM-clusters. We next performed functional enrichment analysis of the DEGs, and in addition to differences in ECM, cell adhesion and other related pathways, we were surprised to find that several immune-related pathways, including T cell activation, cell chemotaxis, Th cell differentiation, cytokine and chemokine activation signaling pathways were significantly enriched. The above results suggest that the significant prognostic difference between distinct ECM-clusters may be due to the tumor immune infiltration traits caused by the distinct ECM characteristics.

Through the ssGSEA, CIBERSORT and ESTIMATE algorithms, we validated the correlation between the ECM-associated prognostic model and immune infiltration characteristics. The ECM-cluster with better prognosis usually exhibit fewer fibroblasts and tumor-promoting cells (such as M2 macrophages and Treg cells), as well as more tumor-killing cells (such as M1 macrophages, NK cells, CD8+ T cells and Th1 cells). Recent studies have also shown that some core components of the ECM are involved in the regulation of immune microenvironment. For example, collagen can affect the function and phenotype of tumor-infiltrating immune cells such as tumor-associated macrophages (TAMs) and T cells (15). The increasing ECM stiffness and aligned arrangement of collagen could not only limit the migration of T cells into the tumor core (28–30), but also affect the interaction between T cells and antigen-presenting cells (APCs) and reduce T cell activation (31, 32). Although studies on the mechanosensing of T cells are still limited, it has also been shown that T cell activation is significantly mitigated when the culture matrix stiffness is increasing (32). In addition, when T cells were cultured on stiff substrates, more anti-inflammatory cytokines were expressed and secreted (31). TAMs are mainly composed of anti-inflammatory M2 macrophages, and the tumor-promoting phenotypes of TAMs are also considered to be affected by the surrounding ECM on the migration and immunosuppressive function of TAMs. Studies have demonstrated that monocytes cultured on the collagen matrix with high density will polarize towards M2 macrophages, and the ability to attract CD8+ T cells and promote T cell proliferation will be significantly reduced (33, 34). Our results undoubtedly solidify and complement the above findings, and provide new evidence and supplement for the link between ECM and immune infiltration.

The CD8+ T cells in TME are of much interest due to their cytotoxic function, while in recent years, CD4+ T cells have attracted more attention for their coordinating role with other immune cells or the direct cytotoxic effects. In our study, we identified a previously unreported link between distinct ECM-clusters and CD4+ Th cells. We found significant differences in the proportion of Th cell subpopulations in breast cancer patients with distinct ECM characteristics. Th1 cells with the function of activating CD8+ cytotoxic T cells increased in the ECM-cluster with better prognosis. While the anti-inflammatory and pro-tumorigenic Th2 cells and immunosuppressive Treg cells were increased in the ECM-cluster with poor prognosis. The immunostimulatory Tfh cells and Th17 cells with dual immune regulatory effects both showed higher abundance in the ECM-cluster with better prognosis. We speculate that the heterogeneity in the expression profiles of Th cells may be partly responsible for the differences in prognosis of distinct ECM-clusters. The reasons for the Th cells heterogeneity caused by distinct ECM characteristics have also been provided by several previous studies. For example, T cells cultured in high density collagen overexpressed the markers of immunosuppressive Treg cells and downregulated markers of cytotoxic T cells (31). Moreover, *in vivo* studies have demonstrated that collagen can promote the increase of CD4/CD8 ratio in infiltrating T cells and CD4+ T cells will differentiate toward pro-tumorigenic Th2 cells (35). The binding of integrin α2β1, a surface marker for Th17 cells, to collagen promoted the synthesis of IL-17, and blocking the binding of collagen and integrin α2β1 decreased the severity of collagen-induced inflammation (36). However, the specific function and mechanism by which ECM regulates Th cells activation and differentiation still need further in-depth studies.

The potential disadvantages of our study should be acknowledged: 1) A total of 4 cohorts (breast cancer cohort from TCGA and 3 GEO cohorts) were included in our study for construction and validation of the ECM-associated prognostic model. However, all the sequencing data and prognostic information were obtained from public databases. We believe that the present prediction model would be more reliable if it can be validated by a prospective clinical trial cohort. 2) Tissue IF staining was applied to verify the link between ECM characteristics and Th cells infiltration, and we will enrich our results with more sufficient research methods in the future. 3) In-depth molecular biology studies will be applied in future studies to elucidate the specific mechanism by which ECM regulated Th cells infiltration characteristics and the prognosis of breast cancer patients.

## Conclusion

In conclusion, we identified and validated the link between ECM characteristics and immune infiltration features in breast cancer, especially the expression profiles of Th cell subpopulations. We also constructed and verified the ECM-associated prognostic model using TCGA and GEO databases, and confirmed that the risk scores of breast cancer patients obtained from our prognostic model were correlated with immune infiltration and TMB. Our findings identify a novel strategy for breast cancer subtyping based on ECM remodeling characterization and reveal the regulatory roles of Th cells in ECM remodeling. Targeting ECM remodeling and Th cells hold potential to be a therapeutic alternative for breast cancer in the future.

## Data availability statement

The datasets presented in this study can be found in online repositories. The names of the repository/repositories and accession number(s) can be found in the article/**Supplementary Material**.

## Ethics statement

The studies involving human participants were reviewed and approved by The Ethics Committee of the First Affiliated Hospital of Xi’an Jiaotong University. The patients/participants provided their written informed consent to participate in this study.

## Author contributions

QT, YYM and BW contributed to the study design and performed experiments. HG and LZZ contributed to data collection. YZ and YWS performed statistical analysis and interpretation. QT and HG drafted the manuscript. All authors contributed to critical revision of the final manuscript.

## Funding

This study was supported by the National Natural Science Foundation of China (No. 82002794) and the Science and Technology Development Incubation Fund of Shaanxi Provincial People’s Hospital (No. 2021YJY-07).

## Acknowledgments

We thank The Cancer Genome Atlas (TCGA) and Gene Expression Omnibus (GEO) for providing transcriptomics and clinicopathological data.

## Conflict of interest

The authors declare that the research was conducted in the absence of any commercial or financial relationships that could be construed as a potential conflict of interest.

## Publisher’s note

All claims expressed in this article are solely those of the authors and do not necessarily represent those of their affiliated organizations, or those of the publisher, the editors and the reviewers. Any product that may be evaluated in this article, or claim that may be made by its manufacturer, is not guaranteed or endorsed by the publisher.

## Supplementary material

Supplementary Figure 1 Overview of expression levels, mutation and CNV of ECM-associated genes in breast cancer. **(A)**. Heatmap showing the differences in expression levels of ECM-associated genes between breast cancer and normal mammary gland tissues. **(B)**. Volcano map showing the distribution of differentially expressed ECM-associated genes between breast cancer and normal mammary gland tissues. **(C)**. Waterfall plot showing the mutation profiles of ECM-associated genes in breast cancer. **(D)**. The CNV profiles of ECM-associated genes in breast cancer. CNV, copy number variation.

Supplementary Figure 2 Identification of ECM-clusters by consensus clustering. **(A)**. The CDF curves obtained by consensus clustering based on ECM-associated genes. **(B)**. The function delta area under CDF curves.

Supplementary Figure 3 The LASSO Cox regression coefficient profiles for establishing the ECM-associated prognostic model. Abbreviations: LASSO, least absolute shrinkage and selection operator.

Supplementary Figure 4 Mutation profiles, stemness index correlation and drug sensitivity prediction of breast cancer patients in different risk groups. **(A–B)**. The mutation profiles of breast cancer patients in high-risk **(A)** and low-risk **(B)** groups. C. Correlation analysis between risk scores and stemness index in breast cancer patients. **(D–G)**. Prediction of drug sensitivity of breast cancer patients to antitumor agents – Bicalutamide **(D)**, CMK **(E)**, Gefitinib **(F)** and Gemcitabine **(G)** - in different risk groups.

Supplementary Table 1 The 328 ECM-associated genes provided by gene ontology terms.

Supplementary Table 2 The information of genes sets for 23 immune cells for ssGSEA algorithm.
