# Supplementary figures and images for: The regulatory roles of T helper cells in distinct extracellular matrix characterization in breast cancer
Source: Front Immunol. 2022 Sep 8;13:871742. doi: 10.3389/fimmu.2022.871742 (PMC9493030; doi:10.3389/fimmu.2022.871742)

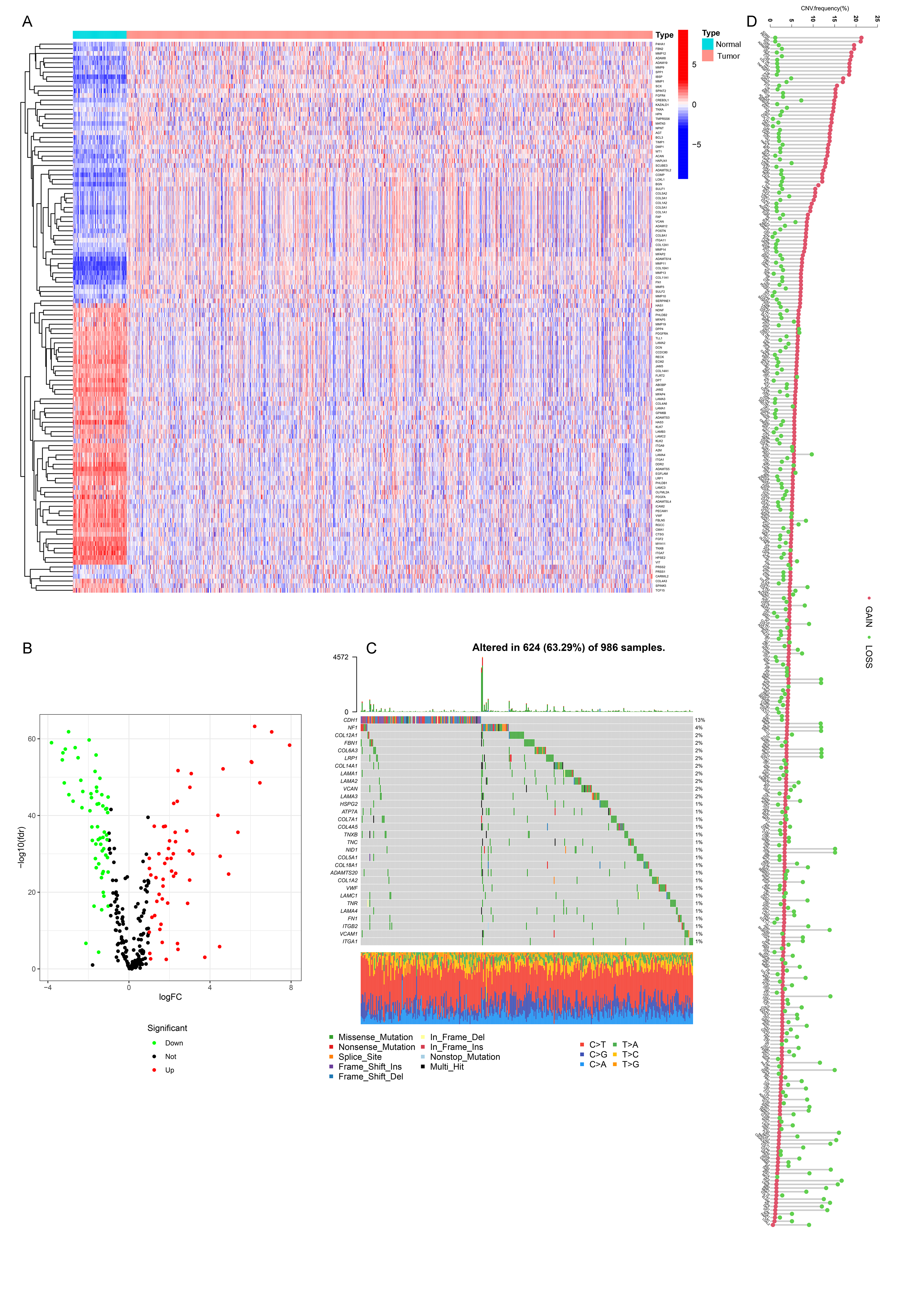

Supplement: Supplementary Figure 1 — Overview of expression levels, mutation and CNV of ECM-associated genes in breast cancer. (A). Heatmap showing the differences in expression levels of ECM-associated genes between breast cancer and normal mammary gland tissues. (B). Volcano map showing the distribution of differentially expressed ECM-associated genes between breast cancer and normal mammary gland tissues. (C). Waterfall plot showing the mutation profiles of ECM-associated genes in breast cancer. (D). The CNV profiles of ECM-associated genes in breast cancer. CNV, copy number variation. [file Image_1.tif]

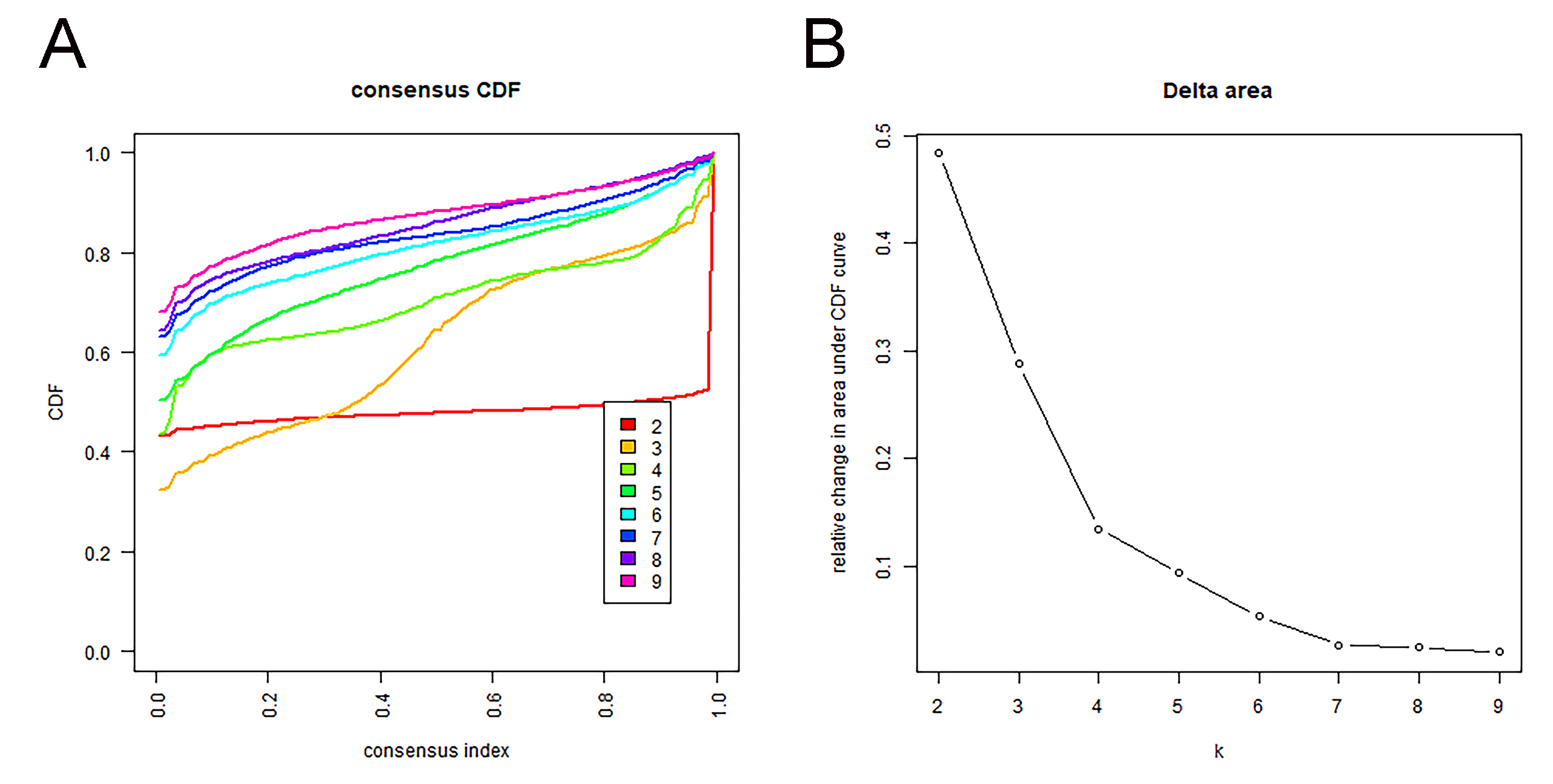

Supplement: Supplementary Figure 2 — Identification of ECM-clusters by consensus clustering. (A). The CDF curves obtained by consensus clustering based on ECM-associated genes. (B). The function delta area under CDF curves. [file Image_2.tif]

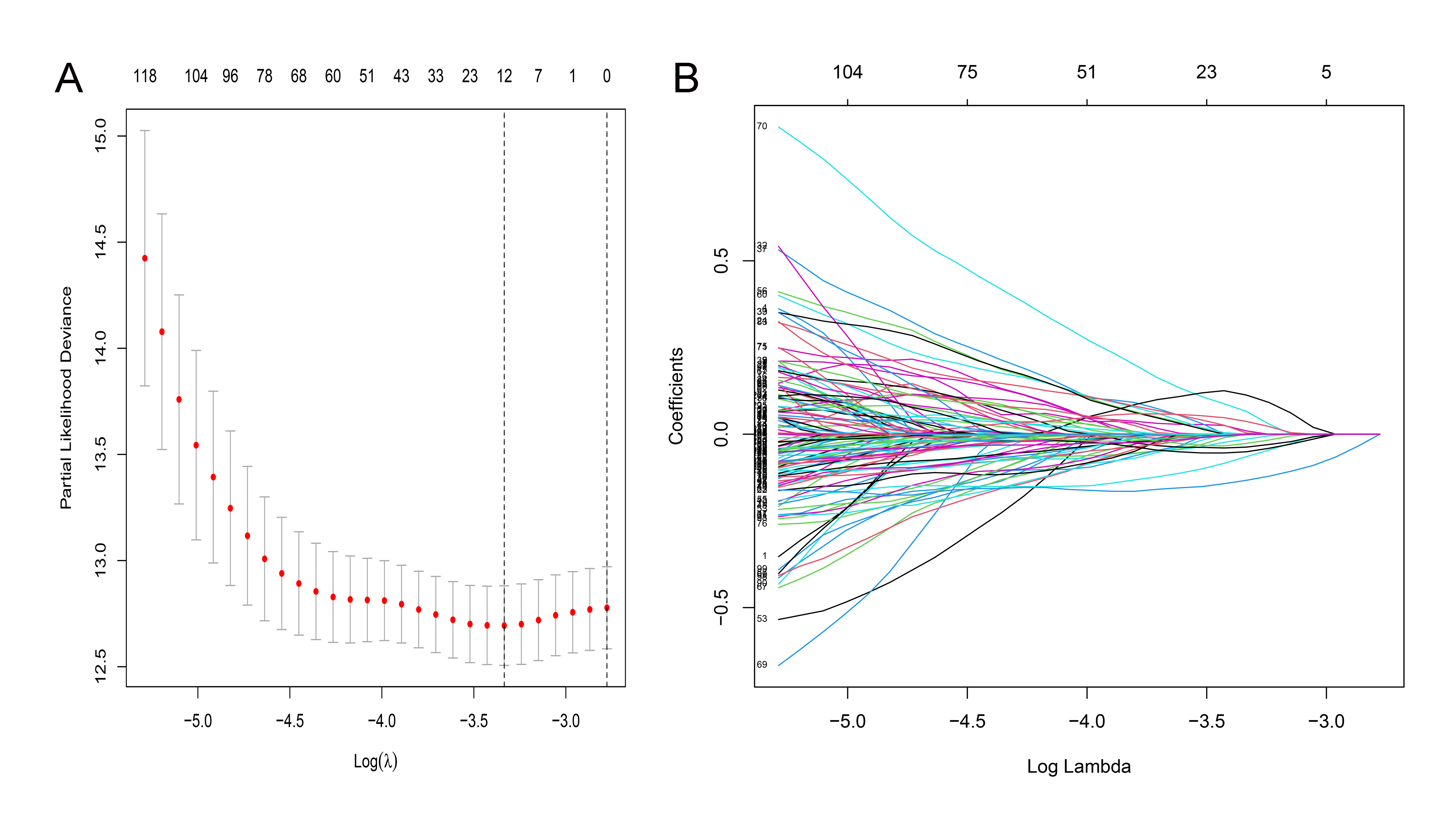

Supplement: Supplementary Figure 3 — The LASSO Cox regression coefficient profiles for establishing the ECM-associated prognostic model. Abbreviations: LASSO, least absolute shrinkage and selection operator. [file Image_3.tif]

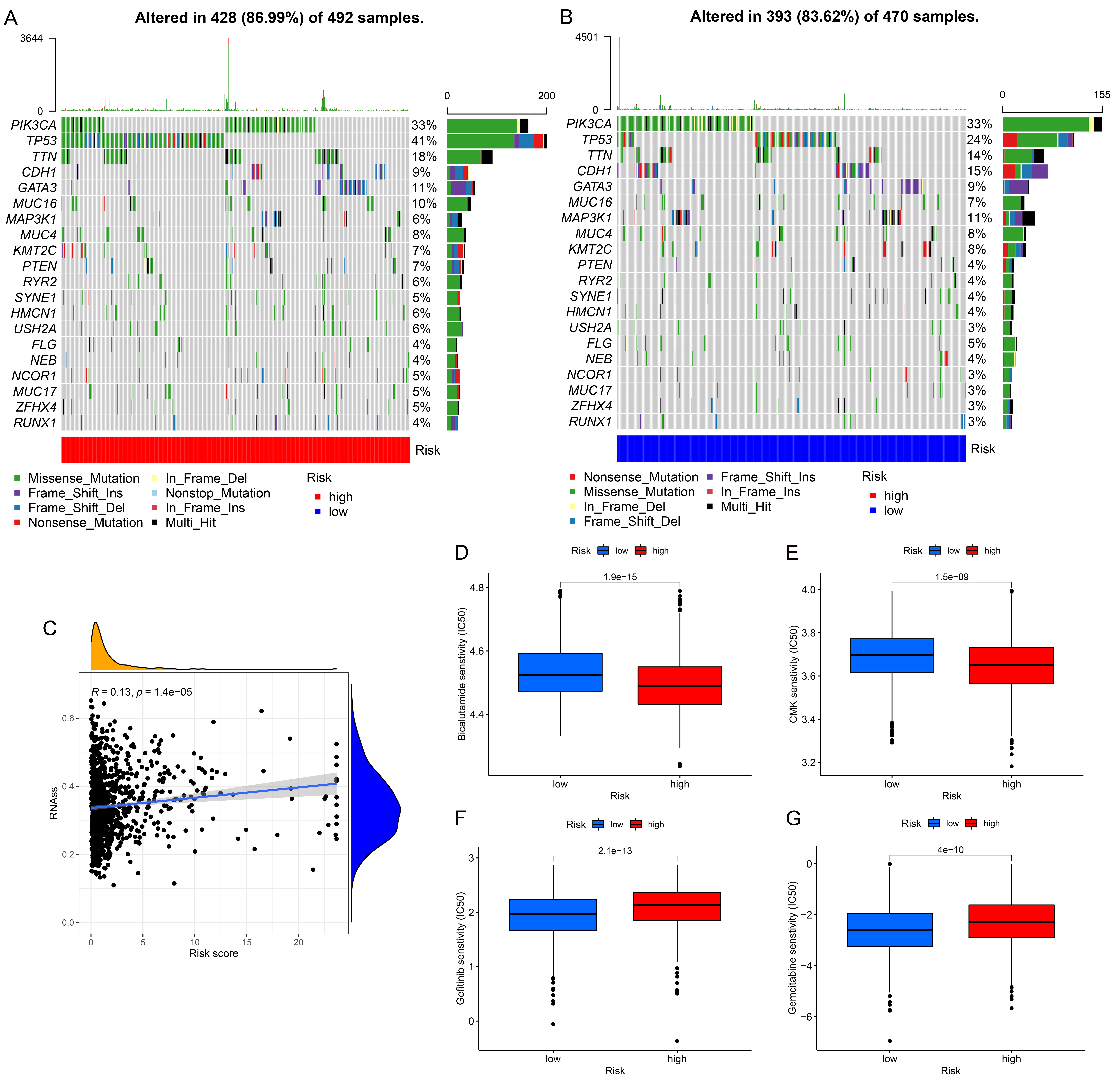

Supplement: Supplementary Figure 4 — Mutation profiles, stemness index correlation and drug sensitivity prediction of breast cancer patients in different risk groups. (A–B). The mutation profiles of breast cancer patients in high-risk (A) and low-risk (B) groups. C. Correlation analysis between risk scores and stemness index in breast cancer patients. (D–G). Prediction of drug sensitivity of breast cancer patients to antitumor agents – Bicalutamide (D), CMK (E), Gefitinib (F) and Gemcitabine (G) - in different risk groups. [file Image_4.tif]
