# Supplementary material for: The regulatory roles of T helper cells in distinct extracellular matrix characterization in breast cancer
Source: Front Immunol. 2022 Sep 8;13:871742. doi: 10.3389/fimmu.2022.871742 (PMC9493030; doi:10.3389/fimmu.2022.871742)
Supplement: Supplementary Table 1 — The 328 ECM-associated genes provided by gene ontology terms. [file Table_1.docx]

Supplementary Table 1. The 328 ECM-associated genes provided by gene ontology terms.

| SCUBE1 | ITGA4 | CST3 | LOXL1 | KAZALD1 | VWA1 | PHLDB2 | ITGA2 |
| --- | --- | --- | --- | --- | --- | --- | --- |
| ECM2 | VCAN | TIMP1 | DDR1 | LUM | THSD4 | LAMB2 | COL5A1 |
| SULF1 | ADAM10 | AGT | CAPN2 | NPNT | TNXB | NFKB2 | LAMC3 |
| SULF2 | ABI3BP | ADAMTS14 | TLL1 | VIT | ITGAX | CTSG | DCN |
| HAS3 | DPT | LAMC1 | CAPN1 | TNF | ITGAL | ACAN | LAMA3 |
| ITGB4 | MELTF | TGFBI | MPV17 | COL27A1 | SPP1 | ITGB6 | CPB2 |
| ITGA6 | DSPP | POMT1 | ANXA2 | WASHC1 | COL19A1 | LAMB3 | ITGA11 |
| PXDN | CD47 | ITGAV | PDGFA | TGFBR1 | GFOD2 | ITGA9 | PRSS2 |
| MMP13 | NOX1 | SOX9 | CMA1 | AGRN | ATP7A | TGFB2 | CYP1B1 |
| ICAM2 | COL8A1 | CYR61 | VTN | ADAM8 | NOXO1 | ELANE | COL6A3 |
| NCAN | HSD17B12 | EGFLAM | CTSV | NID1 | DNAJB6 | NPHP3 | COL6A2 |
| COL9A3 | PTK2 | TLL2 | MATN3 | SH3PXD2B | ADAMTS4 | CLASP1 | COL6A1 |
| COL4A6 | URS000024463E_9606 | SERPINB5 | LAMA5 | GPM6B | ADAM12 | MFAP2 | COL11A1 |
| FBN2 | CRISPLD2 | ADAMTSL4 | ITGA10 | MADCAM1 | FGFR4 | FBLN1 | FSCN1 |
| FBN1 | PDGFB | EXOC8 | COL8A2 | ADAM15 | MATN4 | KIF9 | CTRB2 |
| HAPLN2 | LOX | PDPN | COL18A1 | JAM3 | ADAMTS2 | PECAM1 | HSPG2 |
| BMP1 | HAS2 | CSGALNACT1 | ADAMTS20 | TNR | COL5A3 | MATN1 | FAP |
| MFAP5 | MMP19 | TNFRSF11B | ADAM19 | COL14A1 | IHH | ITGAM | PLOD3 |
| SERPINF2 | COL1A2 | PDGFRA | LAMA4 | ERO1B | ERO1A | HPN | GREM1 |
| ITGA5 | SPOCK2 | HTRA1 | RECK | TGFB1 | SFRP2 | VWF | COL4A1 |
| COL4A5 | COL16A1 | MMP16 | COL5A2 | DAG1 | ELN | MMP12 | COL3A1 |
| ICAM3 | ITGA8 | MMP15 | MMP20 | RAMP2 | CTSS | COL12A1 | COL2A1 |
| CTSL | IBSP | LOXL3 | SPINK5 | ITGB3 | KDR | FOXC2 | COL1A1 |
| FLRT2 | BGN | COL10A1 | RIC8A | BSG | KLK2 | HPSE2 | FERMT1 |
| KLK7 | SCUBE3 | POSTN | DDR2 | MYO1E | SMOC2 | A2M | NPHS1 |
| ICAM1 | TCF15 | ETS1 | SPINT2 | FOXF2 | NDNF | TPSAB1 | LAMA2 |
| CAPNS2 | SERPINH1 | TIMP2 | COMP | FOXF1 | FBLN5 | F11R | THBS1 |
| ADAMTS5 | TTR | CAPNS1 | CTGF | LAMA1 | VPS33B | COL13A1 | LAMB1 |
| RGCC | CD44 | SCX | CLASP2 | COL11A2 | OPTC | HAS1 | VCAM1 |
| ITGAD | JAM2 | TMPRSS6 | SERAC1 | COL9A2 | SMAD3 | CTSK | BCAN |
| COL4A3 | LCP1 | ITGA7 | FLOT1 | EGFL6 | SPINT1 | ITGAE | CTRB1 |
| DMP1 | VIPAS39 | CHADL | CARMIL2 | LAMC2 | PLG | APBB2 | TNXA |
| FGG | CFLAR | NOTCH1 | ICAM5 | PHLDB1 | PRSS1 | LOXL2 | ABL1 |
| FGB | GAS6 | MYF5 | ENG | ITGA2B | NR2E1 | ITGB5 | BCL3 |
| FGA | ITGB2 | ITGB8 | ATXN1L | ELF3 | CREB3L1 | ERCC2 | DPP4 |
| ITGA1 | MMP1 | ITGB7 | MMP8 | MMP9 | ICAM4 | MPZL3 | PRDX4 |
| ADAMTS3 | KLKB1 | ADAMTSL2 | SERPINE1 | COL4A2 | ITGB1 | SH3PXD2A | OLFML2A |
| CDH1 | APP | ITGA3 | FMOD | MMP2 | MMP14 | NF1 | SPARC |
| WT1 | FOXC1 | FURIN | NID2 | MMP3 | LRP1 | COL4A4 | FN1 |
| MMP10 | B4GALT1 | WNT3A | FGF2 | HAPLN1 | P4HA1 | C6orf15 | CCDC80 |
| MMP7 | MYH11 | TNC | ANTXR1 | MFAP4 | COL7A1 | COL9A1 | MMP11 |
